# Supplementary figures and images for: Colonic Coffee Phenols Metabolites, Dihydrocaffeic, Dihydroferulic, and Hydroxyhippuric Acids Protect Hepatic Cells from TNF-α-Induced Inflammation and Oxidative Stress
Source: Int J Mol Sci. 2023 Jan 11;24(2):1440. doi: 10.3390/ijms24021440 (PMC9863622; doi:10.3390/ijms24021440)

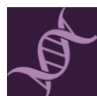

Supplementary Figure S1. Direct effect of DHCA, DHFA and HHA on HepG2 cell viability

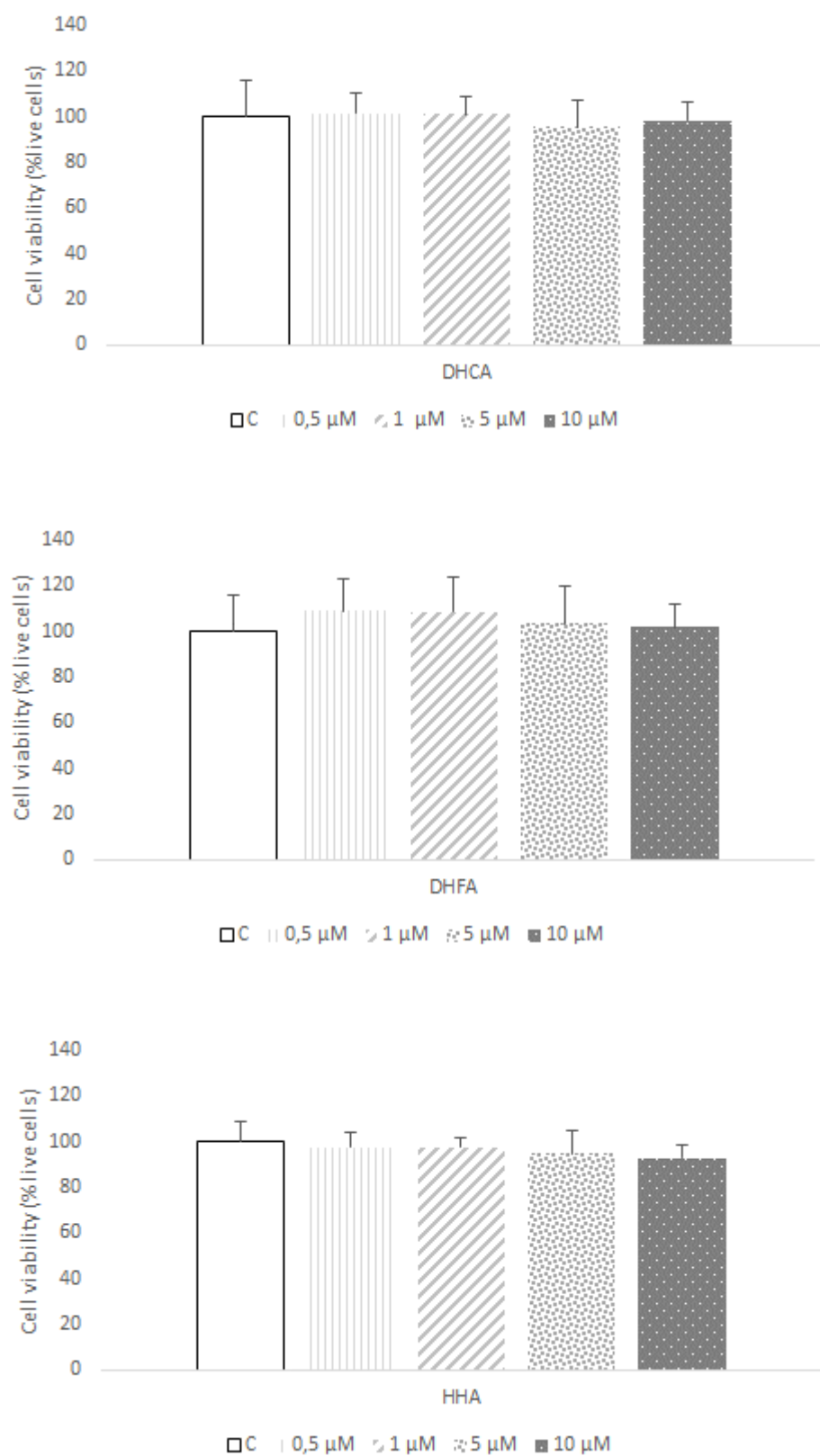

Supplement: Supplementary file 1 [file ijms-24-01440-s001.zip › ijms-2130055-supplementary.pdf]
